# Plant‐derived exosomes extracted from *Lycium barbarum* L. loaded with isoliquiritigenin to promote spinal cord injury repair based on 3D printed bionic scaffold

**DOI:** 10.1002/btm2.10646

**Published:** 2024-01-30

**Authors:** Qilong Wang, Kai Liu, Xia Cao, Wanjin Rong, Wenwan Shi, Qintong Yu, Wenwen Deng, Jiangnan Yu, Ximing Xu

**Affiliations:** ^1^ Department of Pharmaceutics School of Pharmacy, Centre for Nano Drug/Gene Delivery and Tissue Engineering, Jiangsu University Zhenjiang People's Republic of China; ^2^ Medicinal Function Development of New Food Resources Jiangsu Provincial Research Center Zhenjiang People's Republic of China

**Keywords:** 3D bioprinting, M2 polarization, nerve regeneration, plant‐derived exosomes, spinal cord injury

## Abstract

Plant‐derived exosomes (PEs) possess an array of therapeutic properties, including antitumor, antiviral, and anti‐inflammatory capabilities. They are also implicated in defensive responses to pathogenic attacks. Spinal cord injuries (SCIs) regeneration represents a global medical challenge, with appropriate research concentration on three pivotal domains: neural regeneration promotion, inflammation inhibition, and innovation and application of regenerative scaffolds. Unfortunately, the utilization of PE in SCI therapy remains unexplored. Herein, we isolated PE from the traditional Chinese medicinal herb, *Lycium barbarum* L. and discovered their inflammatory inhibition and neuronal differentiation promotion capabilities. Compared with exosomes derived from ectomesenchymal stem cells (EMSCs), PE demonstrated a substantial enhancement in neural differentiation. We encapsulated isoliquiritigenin (ISL)‐loaded plant‐derived exosomes (ISL@PE) from *L. barbarum* L. within a 3D‐printed bionic scaffold. The intricate construct modulated the inflammatory response following SCI, facilitating the restoration of damaged axons and culminating in ameliorated neurological function. This pioneering investigation proposes a novel potential route for insoluble drug delivery via plant exosomes, as well as SCI repair. The institutional animal care and use committee number is UJS‐IACUC‐2020121602.

## 1 INTRODUCTION

Serious complications and an inability to regenerate axons^1^ have been attributed to spinal cord injury (SCI), which is a disease of central nervous system. The progression of SCI can be described in terms of acute and sub‐acute phases.^2^ During acute phase, cytodieresis, necrosis, apoptosis, vascular collapse, and tissue edema may occur in the injured area due to physical stress.^3^ In the sub‐acute phase, the microglia, macrophages, neutrophils infiltrate, and T cells could disrupt barrier of blood–spinal cord and release of many inflammatory factors. This triggers a series of inflammatory effects that cause secondary tissue damage.^4^ Therefore, regulation of this inflammatory response and promotion of nerve regeneration have become the main strategies to be used for SCI repair.

The use of bionic scaffolds has taken SCIs repair in a new therapeutic direction with tissue engineering technology development. Namely, 3D‐printed scaffolds can be feasibly individualized and scaled to the shape and length of any patient‐specific lesion.^5^, ^6^, ^7^ Implementing this bionic scaffold with good biocompatibility and appropriate mechanical properties could facilitate spinal cord repair by controlling drug release, promoting synaptic regeneration, and reducing scar formation.^8^ Thus, the implantation of bionic scaffolds with therapeutic properties holds great promise for the repair of SCIs.^9^, ^10^

Exosomes are being widely explored as drug carriers.^11^, ^12^, ^13^ It has been demonstrated that animal‐derived exosomes can effectively stimulate angiogenesis^14^ and alleviate inflammation and oxidation, thereby promoting recovery of motor function and improving the microenvironment after SCI.^15^, ^16^, ^17^ Compared to animal‐derived exosomes, plant‐derived exosomes (PEs) are a class of naturally occurring lipid bilayer extracellular vesicles used for material and information exchange between plant cells, which are approximately 50–150 nm in diameter.^18^ It has been demonstrated that PEs exhibit a lot of beneficial effects, viz., antiviral, anti‐inflammatory, and antitumor properties, wherein they are involved in defense responses against pathogenic attacks.^19^, ^20^, ^21^ Compared with the successful application of mammalian‐derived exosomes in SCI repair,^22^, ^23^ no such research on PEs has been reported so far. *Lycium barbarum* L. is a homologue of medicinal and food plants which belong to *Lycium* genus in Solanaceae family, amid principal distribution in northwest China.^24^ The fruit of *L. barbarum* has hepatoprotective,^25^ anti‐radiation,^26^ antioxidant and immune‐enhancing,^27^ anti‐inflammatory, and anti‐Alzheimer's effects.^28^ The anti‐inflammatory and antioxidant activities can interfere with the inflammatory balance in vivo, which would be an important strategy for SCI treatment. Nevertheless, the use of exosomes derived from *L. barbarum* has not been previously reported.

Isoliquiritin (ISL), a flavonoid derived from licorice,^29^ inhibits lipopolysaccharide‐stimulated expression of cyclooxygenase‐2 (COX‐2) and inducible nitric‐oxide synthase (iNOS) and decreases tumor necrosis factor‐alpha (TNF‐α) and interleukin‐6 (IL‐6) release.^30^ In addition, ISL has neuroprotective effects in damaged PC12 cells.^31^ However, since ISL is insoluble in water, there are challenges in increasing its solubility and improving its pharmacological activity.^1^, ^32^

We designed and 3D bioprinted a bionic scaffold containing ISL encapsulated within PEs from *L. barbarum* with the aim to increase ISL solubilization and enhance its anti‐inflammatory and neuronal differentiation properties.

## 2 MATERIALS AND METHODS

### 2.1 Experimental materials

Hebei Rishengchang modern agricultural development Co. Ltd. supplied fresh *L. barbarum*. High‐glucose DMEM, DMEM/F12, GlutaMAX™, fetal bovine serum and MEM non‐essential amino acid solution (100×), N2, B27 were provided by Thermo Fisher Technology (China) Co., Ltd. Recombinant human HB‐EGF, recombinant human fibroblast growth factor (FGF) basic, and poly‐l‐lysine were purchased from PeproTech, Inc. High‐performance RIPA lysis solution (tissue/cell), PMSF, DAPI, and protein loading buffer (with DTT) were supplied by Beijing Solabio Life Sciences Technology Co., Ltd. Shanghai Yaenzyme Biotechnology Co., Ltd. provided tricolor pre‐stained protein marker (10–250 kDa), bicinchoninic acid (BCA) assay kit, and PAGE Gel Rapid Preparation Kit. The following rabbit‐derived antibodies (Anti‐beta III Tuj1, Anti‐Sox2, AntiArg‐1, Anti‐Nestin, Anti‐GFAP, Anti‐GAP43, Anti‐MAP2, Anti‐MBP, Anti‐iNOS, Anti‐CD206, Anti‐AKT, and Anti‐pAKT) were purchased from Abcam Plc. HRP‐conjugated Affinipure goat anti‐rabbit IgG (H + L) and Fluor488 and 594‐conjugated goat anti‐rabbit IgGs (H + L) were obtained from Affinity. The ready‐to‐use rabbit IgG‐immunohistochemistry kit (SABC) was provided by Boster Biological Technology. Labgic Technology Co., Ltd. supplied bovine serum albumin, reactive oxygen kit, and glycine. Rat IL 1β, IL 6, IL 10, and TNF‐α ELISA kits were purchased from Invitrogen Thermo Fisher Technology (China) Co., Ltd. Sigma Aldrich (Shanghai) Trading Co., Ltd. provided other materials that were used in this work.

### 2.2 Extraction and purification of exosomes

#### 2.2.1 Plant‐derived exosomes

The fresh *L. barbarum* was washed with double‐distilled water, and added to an appropriate amount of phosphate‐buffered solution (PBS), before it was placed in a homogenizer to make a homogenate. We immediately added a protease inhibitor to the homogenate after its filtration. Afterwards, we adjusted pH of the homogenate to 7 using 1 mol/L Tris–HCl. Later on, centrifugation of homogenate was carried out for 20 min at 400, 800, and 15,000×*g* for 20 min, as well as 1 h at 100,000×*g*. Afterwards, we collected the precipitate before resuspension in Tris–HCl (20 mmol/L). We then vortexed the resuspended precipitate thoroughly to obtain crude extracted PEs from *L. barbarum*.

The crude PEs were further purified using sucrose density gradient centrifugation. Specifically, the crude PEs were transferred to 30%, 45%, and 60% solutions of sucrose prior to 2 h of centrifugation at 150,000×*g*. We collected an intermediate layer of 30%–45% sucrose solution prior to addition of an equal amount of PBS and centrifugation of the solution for 1 h at 150,000×*g*. The PEs were collected, washed, and suspended in 1 mL of PBS. The purified PE solution was obtained by filtering through 0.22 μm filter before storage at 4°C.

#### 2.2.2 Mesenchymal stem cells‐derived exosomes

Mesenchymal stem cells (MSCs)‐derived exosomes (ME) were isolated using standard protocols.^33^ Briefly, MSCs was isolated and cultured until they reach 70%–80% confluency. The culture medium was collected and centrifuged at 300×*g* for 10 min to remove any cells or debris. The supernatant was transferred to a new tube and centrifuged at 2000×*g* for 10 min to remove larger vesicles. The resulting supernatant was centrifuged at 10,000×*g* for 30 min to pellet the exosomes. The obtained ME can be resuspended in PBS and was storage at −80°C.

### 2.3 Characterization of exosomes

#### 2.3.1 Morphological characterization

Before dropping onto a carbon‐coated copper sheet, we dehumidified the purified PE solution for 10 min. Washing with double‐distilled water removed excess stain after negative staining of the copper sheet with phosphotungstic acid. Hitachi 7500 transmission electron microscope (TEM) operating at 80 kV was employed to observe and record PE morphology. Later, we resuspended PE in PBS solution prior to filtration via a filter (0.22 μm). A particle size analyzer (Malvern Panalytical) was used to determine the size and zeta potential of PE particles through dynamic light scattering (DLS) at 25°C.

#### 2.3.2 Protein characterization

In brief, we determined protein concentrations in PEs using a BCA kit. Standard protein concentrations of 0, 1, 2, 4, 8, 12, 16, and 20 μL were prepared according to assay protocol and mixed with the BCA solution. A microplate reader was used to measure absorbance of each well at 562 nm to generate a standard curve. The absorbance of PE solution was measured and calculated with the standard curve.

### 2.4 Drug loading of PEs

Mixing of ISL (0.1 mg/mL final concentration) with PEs was accomplished with ultrasound for 15 min at 35 kHz and 4°C. The ultrasonicated exosomes now comprising ISL were purified by ultrafiltration through 100 kDa filters (Amicon, Millipore) and washed 10 times with PBS to obtain ISL encapsulated by PEs (ISL@PE). High performance liquid chromatography (HPLC) was used to determine the final concentration of ISL in the formulation.

### 2.5 In vitro release of ISL from ISL@PE

Dynamic dialysis was used to perform the in vitro release experiments. The release behaviors were simulated in a normal human body fluid environment (pH 7.4 PBS solution), inflammatory environment (pH 6.8 PBS solution), and gastric fluid environment (pH 1.2 HCl solution). Two milliliters of ISL@PE and ISL were measured precisely before they were placed in separate pretreated dialysis bags. We then tied tightly at both ends, and placed in 30 mL of a release medium containing different media. The release medium was shaken at 100 rpm for 48 h at 37°C, while 2 mL of release medium was aspirated at 0, 0.5, 1, 2, 4, 6, 8, 12, 24, 36, and 48 h, accordingly. To maintain the sink condition, we replenished the system with same and equal volume of release medium, while the content of ISL in the release medium aspirate was determined with HPLC after filtration via a filter (0.45 μm). The cumulative release was calculated in triplicate for each sample.

### 2.6 Characterization of 3D printed ISL@PE in hydrogel

The HE stained spinal cord T9 segment was scanned, and a 3D mimic of the spinal cord segment was designed using SolidWorks software accordingly. The designed model was cut with a slicer into G‐codes and printed using a 3D bioprinter (RegenHu Discovery). Then, gelatin methacryloyl (GelMA, 5%), lithium phenyl‐2,4,6‐trimethyl‐benzoyl‐phosphinate (LAP, 1%), and ISL@PE (5 mg/mL) were mixed to be used as bioink. The complex hydrogel was then phosphorylated at 405 nm for 30 s. Scanning electron microscopy (SEM) was used to examine the morphology of the hydrogel containing ISL@PE. The Young's modulus of different concentration of GelMA (3%, 5%, 7%, and 10%) was measured using a universal testing machine to illustrate the mechanical properties. The ISL@PE in hydrogel was placed in dissolution tester at varied pH values (namely 1.2, 6.8, and 7.4), while the cumulative release of ISL was detected at 0, 0.25, 0.5, 1, 2, 4, 8, and 12 h and 1, 2, 3, 4, 6, 8, 10, 12, 14, and 16 days.

### 2.7 Cell cultivation

#### 2.7.1 Neural stem cells (NSCs) isolation and culturing

Brain tissue was separated from the fetal SD rats, dissected, and cultivated in a medium comprising DMEM/F12 (1:1) as well as B27 (1%), bFGF (20 ng/mL), EGF (20 ng/mL), MEM‐NEAA (1%), N_2_ (1%), penicillin and streptomycin (PS, 1%). Culturing of the entire cells was carried out for 7 days, while neurospheres were observed. Later on, we collected the neurospheres and placed them in a polyline pretreated plate for the following steps.

#### 2.7.2 Cultivation of N9 microglial cells

High glucose DMEM medium comprising fetal bovine serum (10%) was used to culture the N9 microglial cell line at 5% CO_2_ and 37°C. When the confluence reached 90%, we digested the N9 cells with trypsin before passaging for continuous cultivation.

### 2.8 Identification of rat NSCs

Fixation of neurospheres with 4% paraformaldehyde (PFA) was performed for 15 min. Using PBS, we washed (twice) the fixed neurospheres prior to incubation with a block buffer (BB, 5% bovine serum albumin and 0.3% Triton X‐100) at 4°C overnight. The BB solution was replaced with various rabbit primary antibodies, including GFAP, Nestin, Sox2, and Tuj1 (1:100 dilution) at 4°C. As stated above, washing of the neurospheres with PBS after 12 h was carried out before 12 h of incubation with Fluor488‐conjugated sheep anti‐rabbit secondary antibodies (1:100 dilution) at 4°C. Likewise, we washed the neurospheres with PBS before 15 min of staining with DAPI (500 ng/mL) at 25°C, and washing with PBS once again. Observation of the stained spheres was accomplished under a fluorescence microscopic system.

### 2.9 Effect of ISL@PE on neural differentiation

The neurospheres were seeded in a 24‐well plate. Free ISL, PE, ME, and ISL@PE were added to each well and co‐cultivated for 4 days. Each well was immunostained with rabbit primary antibodies against Sox2, Tuj1, and MAP2. Observation of all the cells was carried out under a microscope.

The time–effect relationship of the induced synaptic growth of NSCs was measured separately. Later, 24 h cultivation of NSCs was accomplished in 24‐well plates, while free ISL, PE, ME, and ISL@PE were added into each well. Synaptic growth was observed under a phase‐contrast inverted microscope (Ti‐U) and photographed at 24, 36, and 48 h. Synaptic growth cells were determined as cells with a synaptic length more than two times the cell diameter. Afterwards, we calculated the percentage of synaptic growth cells as percentage of total 300–400 cells in each well, wherein they were considered synaptic growth cells.

### 2.10 Modulation of LPS‐treated microglia by ISL@PE

Seeding of N9 microglial cells was carried out in a 24‐well plate before division of the cells into five groups: normal, lipopolysaccharide (LPS), LPS + free ISL, LPS + PE, LPS + ME, and LPS + ISL@PE. The LPS group was stimulated with LPS (1 μg/mL) for 24 h to establish an inflammatory model of N9 cells, while free ISL, PE, MSCs exosomes, and ISL@PE groups were incubated with LPS‐treated N9 cells after 24 h. The proportion of CD206‐, iNOS‐, and Arg‐1‐positive N9 cells was measured with immunofluorescence, while the active oxygen content was detected by flow cytometry. Using reactive oxygen species (ROS), we measured levels of ROS with a ROS kit. The protein expressions of iNOS, CD206, Arg‐1, AKT, and pAKT were appropriately detected by western blot.

### 2.11 3D‐printed scaffolds for application of in vivo SCI repair

The SD rats (male, 200 g) were adaptively fed for 7 days and divided into sham‐operated, SCI model, 3D printed hydrogel with free ISL, and 3D printed hydrogel with ISL@PE groups, with each group comprised of five rats. Aesthesis of the rats with 30 mg/kg of intraperitoneal pentobarbital sodium was carried out. The spinal cord was exposed by removing the T9 vertebral plate to open the spinal canal before removal of the spinal cord (2 mm) and suturing of muscle and skin layers. In sham‐operated group, we exposed the spinal cord by removing only the T9 vertebral plate, while the rest of the procedure was the same as that in the SCI rat model. The rats in 3D printed groups were implanted with a GelMA hydrogel scaffold containing free ISL, or a GelMA hydrogel scaffold containing ISL@PE. All the rats were injected with penicillin to prevent infection within 7 days of the operation, while a daily urination massage was administered until their bladder urination function was recovered.

### 2.12 Hindlimb motor function assessment in rats via behavioral scoring

After 8 weeks of treatment, we weekly measured inclined plane test, Basso, Beattie, and Bresnahan (BBB) scores, and open field test of SD rats in different groups. We divided the entire rats into four groups, namely sham, model, 3D printed hydrogel with ISL, and 3D printed hydrogel with ISL@PE groups. For the BBB scores, different groups of rats were placed one by one on a wide table and allowed to crawl freely. The rats were monitored by two observers who were unaware of the grouping. Range of motion of the king joint of the hindlimb, movement of the heart, and coordination between the hindlimb and forelimbs were observed. Scoring of the hindlimb motor function of each rat was done according to the BBB scoring system.^34^ We performed inclined plane test by placing each group of rats at different times (1–8 weeks) one by one on an inclined plate using a rubber mat. Afterwards, we placed the longitudinal axis of the rat's body parallel to longitudinal axis of inclined plate. Meanwhile, we placed the rat's head toward the elevated side of inclined plate, while we observed the maximum angle at which the rat could stay on the inclined plate for 5 s at an angle of 5° from 0° each time it was elevated.^35^ The open field experiment was performed by placing rats in a 100 cm × 100 cm open field for 5 min of free movement, and counting their total distance traveled were recorded and analyzed using a computer.^36^

### 2.13 Morphology quantification

To assess spinal cord regeneration, HE, Nissl, fluorescent, and IHC staining procedures were used and quantified. After 8 weeks, we collected the injury site of spinal cord in each group before fixation with 4% PFA. After paraffin sectioning, the slices were subjected to HE, Nissl, immunofluorescent, and immunohistochemical staining methods. The immunohistochemical results were quantified using Fiji software (1.53f51).

### 2.14 ELISA detection of injured spinal cord with 3D printed hydrogel with ISL@PE

After behavioral scoring for 8 weeks, we sampled blood (5 mL) from the abdominal aorta of the rats prior to 10 min of centrifugation at 3000 rpm at 4°C. Later, we aspirated the upper serum layer. Using ELISA kits for IL‐(1β, ‐4, ‐6, and ‐10), we accordingly measured the serum levels of these cytokines based on their respective protocols.

### 2.15 Western blot detection

Through BCA assaying method, we determined the protein quality and concentration in the supernatant, which collected from the spinal cord tissue with injured segments in each group that had been treated for 8 weeks. Separation of equal amounts of proteins was accomplished with sodium‐dodecyl sulfate‐polyacrylamide gel‐electrophoresis (SDS‐PAGE). We transferred the proteins to poly‐vinylidene fluoride (PVDF) membranes before we closed in the closure solution. Later, we added different primary antibodies derived from rabbit, namely anti‐GFAP (1:300), anti‐GAP43 (1:800), anti‐NF200 (1:200), and anti‐MBP (1:300). After overnight incubation at 4°C, we washed the membrane with PBST before addition of secondary antibodies and 12 h of incubation at 4°C. Staining of the membrane was accomplished with enhanced chemiluminescence (ECL) prior to scanning and grayscale analysis.

### 2.16 Statistical analysis

Data analysis was accomplished with SPSS 21.0 software (SPSS Inc., Chicago, IL, USA), wherein mean ± standard deviation (*x* ± *s*) was used to express the data. Comparison of two groups was done with *t*‐tests while, comparison of three groups was accomplished with one‐way ANOVA. Statistically, consideration was given to *p* < 0.05 as significant level.

## 3 RESULTS AND DISCUSSION

### 3.1 PEs identification and characterization

**FIGURE 1:**
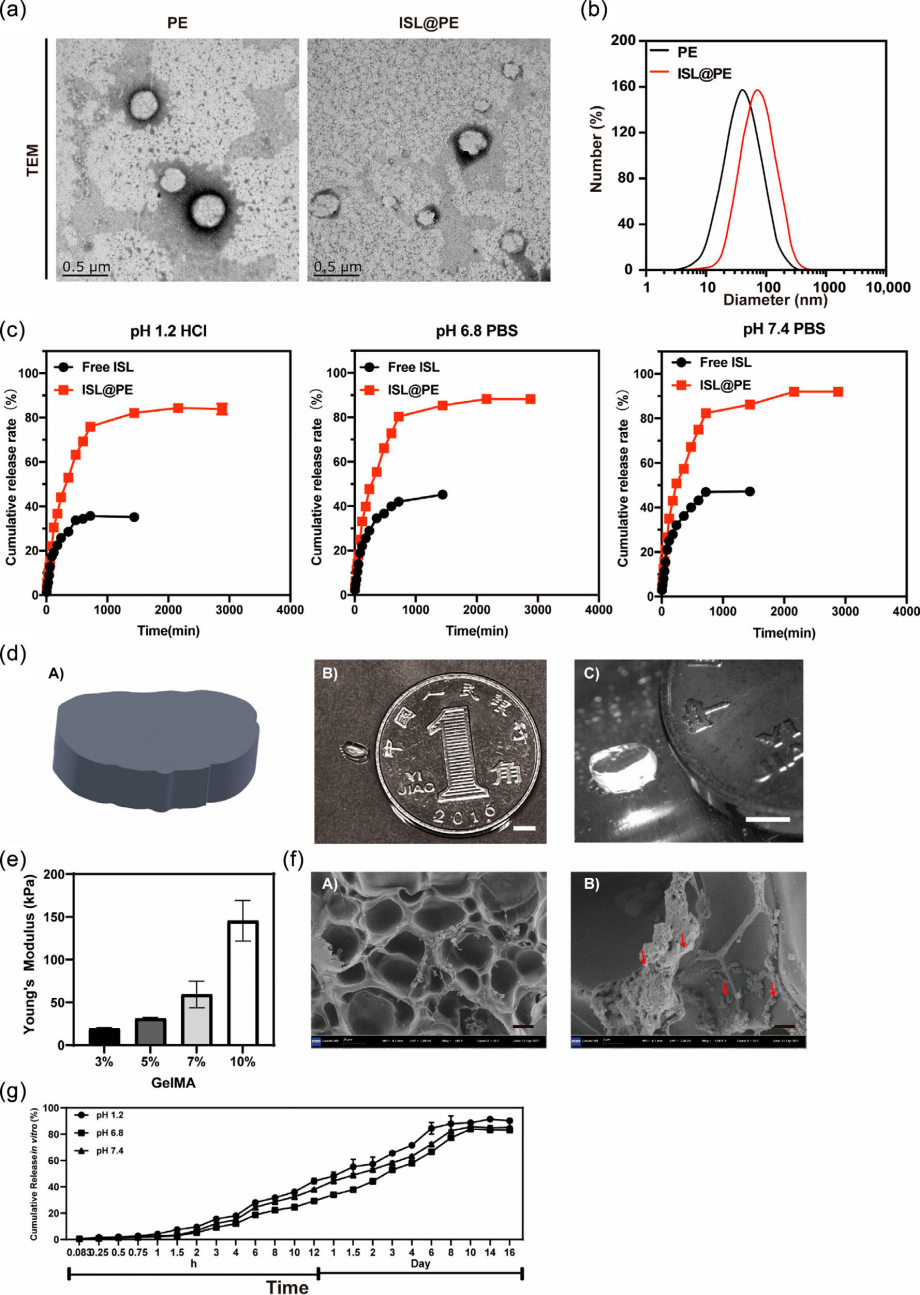
Characterization of plant‐derived exosomes (PE) and isoliquiritigenin (ISL)‐loaded plant‐derived exosomes (ISL@PE) in hydrogel. (a) Transmission electron micrographs of PE and ISL@PE. (b) The size distribution of PE and ISL@PE. (c) The in vitro cumulative release rate of ISL@PE and free ISL at pH 1.2, 6.8, and 7.4. (d) 3D‐printed spinal cord hydrogel scaffold. (A) Model design of mimic spinal cord segment. (B, C) 3D‐printed hydrogel (scale bar equals to 3 mm). (e) Young's modulus of GelMA. (f) The scanning electron micrographs of the 3D‐printed functional hydrogel (scale bar equals to 20 μm). The arrows indicate the ISL@PE. (g) The in vitro release of ISL@PE in hydrogel under various pH conditions.

In terms of morphological structure, PE is similar to exosomes of animals, which is a phospholipid bilayer.^37^ The identification of PEs is mainly based on the observation of morphology by TEM, while DLS was used to analyze particle size distribution. In the present study, three layers of PE were collected after sucrose density gradient centrifugation, wherein the upper and middle layers were combined and weighed after lyophilization. The TEM results showed that PEs had a typical round or oval cup‐shaped lipid bilayer structure (Figure 1a) with a size of 151.45 ± 3.86 nm (Figure 1b), and a zeta potential of −5.34 ± 0.472 mV. The particle distribution index (PDI) of the sample was 0.147 ± 0.0135. These results showed that the purified PE solution was homogeneous in morphology and consistent with the microscopic identification of exosomes, which were a disc‐shaped vesicle‐like structure with an obvious outer bilayer.^38^ Based on the morphology and size analysis, we verified that natural nanovesicles (PE) were successfully extracted from *L. barbarum* L.

### 3.2 In vitro characterization of ISL@PE

Compared to traditional nanocarrier systems, PEs are biocompatible, highly practical, safe, and non‐toxic and can successfully carry model drugs and target genes for further application in the study of drug delivery systems for various diseases.^39^ Exosomes possess a lipid bilayer membrane structure that can surround hydrophobic drugs and deliver them to recipient cells.^40^, ^41^ In this study, PEs were used as a carrier to entrap ISL to form ISL encapsulated PE (ISL@PE), while free ISL was used as a control group. The size of an ISL@PE was 143.7 ± 5.36 nm (Figure 1b), while the PDI was 0.58 ± 0.18. The drug loading capacity and encapsulation rate were 13.5 ± 0.27% and 82.6 ± 4.19%, respectively. As shown in Figure 1c, at pH 6.8, the cumulative release rates of free ISL at 4 and 48 h were 14.9% and 35.4%, respectively, while those of ISL@PE at 4 and 48 h were respectively 15.4% and 80.57%. Similar trends were observed at pH 1.2 and 7.4. This indicated that the ISL@PE exhibited significantly slower release properties than the free ISL.

### 3.3 3D printed hydrogel with ISL@PE

GelMA hydrogels can be modified in medicine to incorporate bioactive substances such as factors or cells and provide an excellent environment for cell growth.^42^ Specifically, 3D‐printed GelMA hydrogels have a three‐dimensional mesh structure and high‐water content that mimics the mechanical properties of natural tissues.^43^, ^44^ A 3D printing needle with a diameter of 260 μm was used to print a 4 mm × 2 mm × 2 mm spinal cord‐like scaffold according to the design model seen in Figure 1dA. The printed hydrogel scaffold was shown in Figure 1dB,C. We measured the Young's modulus of different concentration of GelMA and found the Young's modulus increased (from 20 to 140 kPa) with the increased concentration of GelMA (from 3% to 5%), which was shown in Figure 1e. According to previous studies,^45^ the Young's modulus of the native spinal cord tissue ranges from 10 to 100 kPa. Therefore, our 5% GelMA scaffold has a comparable stiffness to the spinal cord tissue and may have a better biocompatibility and integration than other scaffolds. However, the mechanical properties of GelMA scaffolds can be further improved by various methods.^46^ Therefore, the mechanical properties of the bionic scaffold should be considered in future studies, and we should discuss the potential challenges and solutions for achieving better clinical outcomes. We characterized morphology of ISL@PE‐containing hydrogel with SEM, wherein the lyophilized scaffold exhibited a porous structure as shown in Figure 1fA. The ISL@PE in the scaffold were visible under further magnification (red arrows in Figure 1fB). The in vitro release results of ISL@PE in the gel showed a significant control increase of ISL in the hydrogel (Figure 1g), thereby presenting a controlled release for 16 days at different pH conditions. This significantly slow‐release effect was attributed to the reticulated cross‐linked structure of the hydrogel which contained ISL@PE.^47^ Thus, the release of ISL from the hydrogel can be controlled for a longer time compared to ISL@PE without hydrogel, even under low pH conditions.

### 3.4 Identification and NSCs differentiation of ISL@PE

**FIGURE 2:**
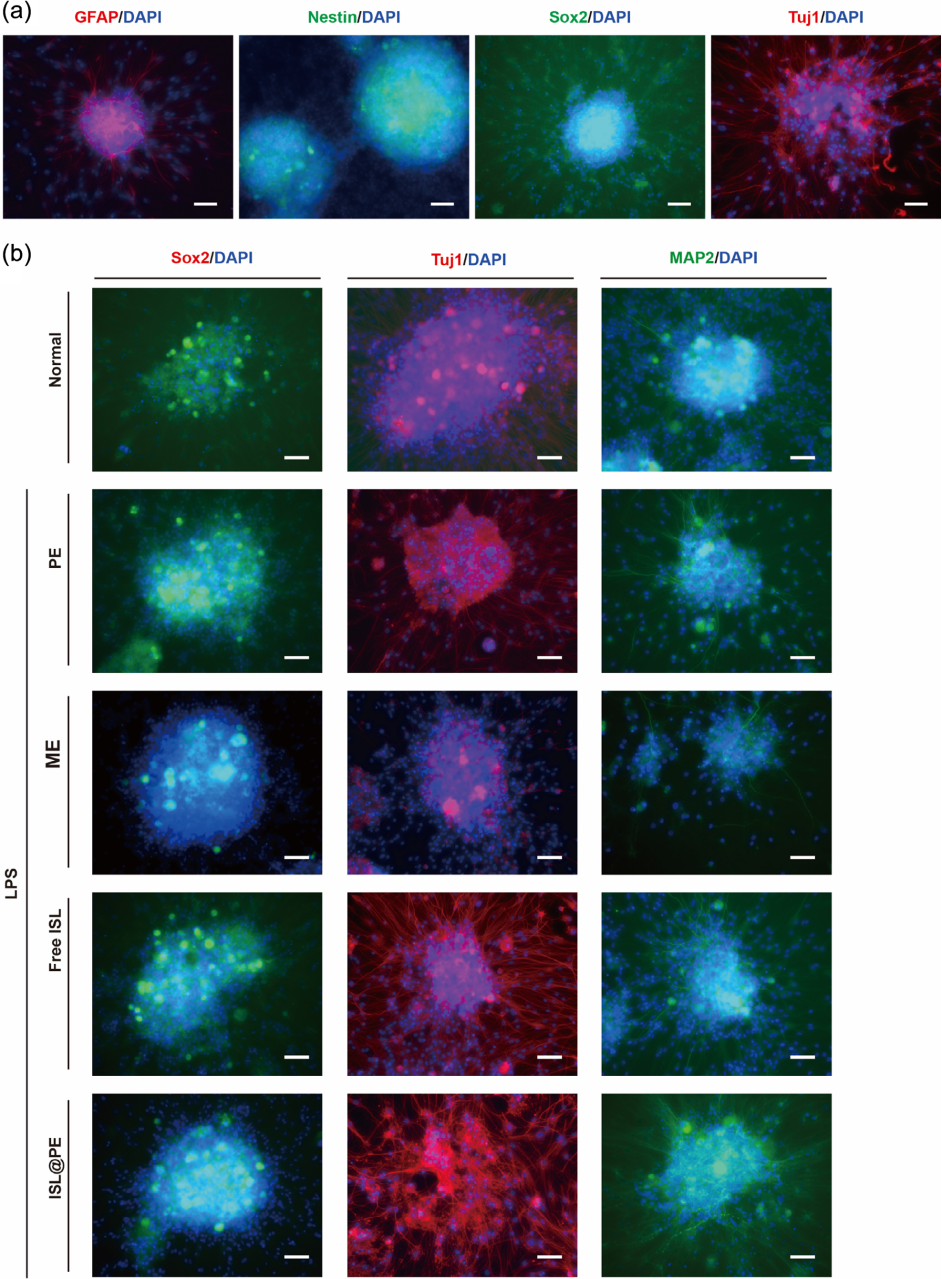
Neuro differentiation effect of isoliquiritigenin (ISL)‐loaded plant‐derived exosomes (ISL@PE). (a) Fluorescent staining of neural stem cells expressing GFAP, Nestin, Sox2, and Tuj1. (b) Fluorescent staining of neural stem cells expressing Sox2, Tuj1, and MAP2 in control, PE, ME, free ISL, and ISL@PE formulations. Scale bar equals to 50 μm.

We collected neurospheres from fetal rats and identified them using immunostaining. The cells stained positive for Nestin, Sox2, Tuj1, and GFAP proteins (Figure 2a). Nestin is the main backbone protein of mammalian NSCs^48^ that gradually disappears with cell differentiation. Biologically, Nestin is specific NSCs marker, which is one of the main indicators used for stem cell identification. Aside being a key transcription factor for regulation of self‐replication and pluripotency of stem cells, Sox2 also regulates the proliferation of NSCs.^49^ GFAP was chosen to identify the multidirectional differentiation ability of NSCs.^50^ As a principal constituent of the axonal cytoskeleton, β III Tuj1 is widely used as a marker to distinguish neurons from other cell types.^51^ Thus, a positive staining indicated that the cultured cells were NSCs.

### 3.5 Neuro differentiation of ISL@PE

**FIGURE 3:**
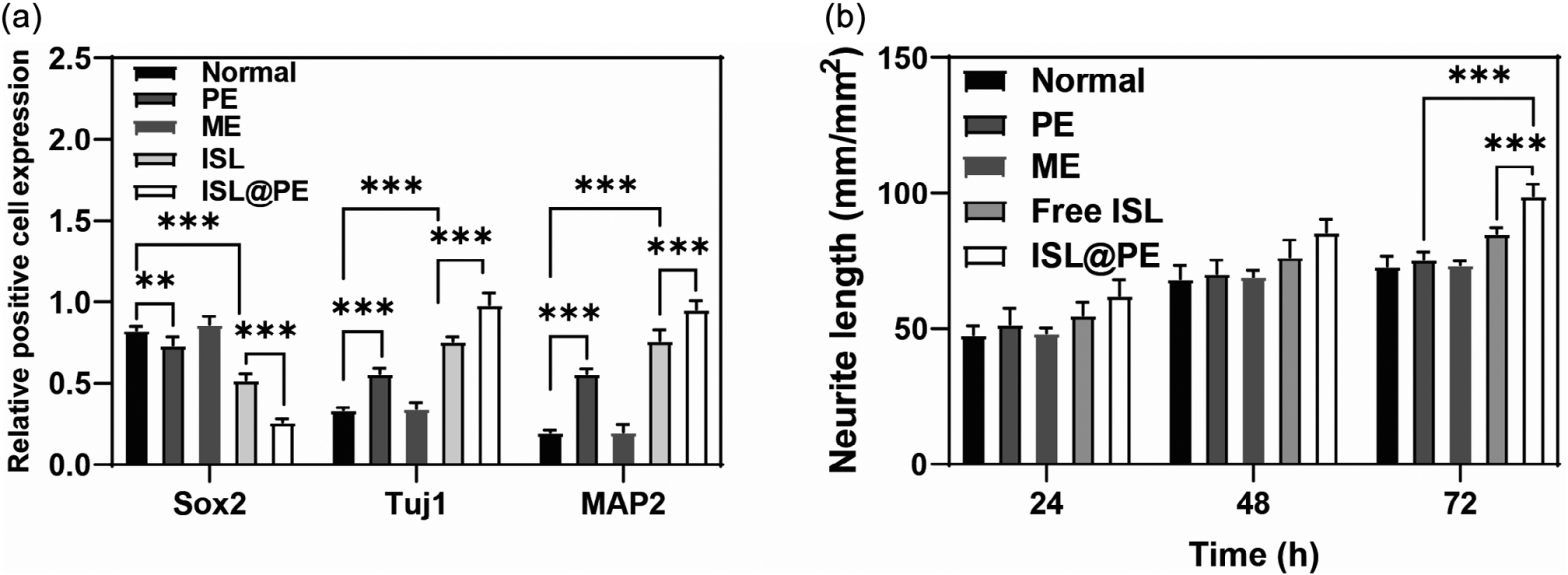
Quantitative analysis of neuro differentiation effect of ISL@PE. (a) Quantitative analysis of relative positive cell expressed in immune fluorescent staining. (b) Neurite length of differentiated neural stem cells in different groups. *p > 0.05; **p < 0.05; ***p < 0.01.

To compare ISL@PE effects on differentiation ability of NSCs, the expression of mature neuronal markers (Map2 and Tuj1) was examined. As shown in Figure 2b, both proteins were positively expressed in the PE, ME, ISL, and ISL@PE groups comparable to normal batch. It was discovered in Figure 3a that levels of Tuj1 and MAP2 relative expression in ISL@PE batch increased substantially compared to that of ISL, PE and ME groups (*p* < 0.01). Contrarily, Sox2 protein expression in control group increased markedly compared to other groups (*p* < 0.01). ME group showed no changes with normal group. This indicates that both ISL and PE can induce mature neuronal differentiation. Thus, the ISL@PE group demonstrated a strong combined effect.

Synaptic growth is not only an inevitable process and an important feature of neural plasticity reconstruction during nerve injury repair but is also essential for nerve cell localization and establishment of synaptic connections during neural development.^52^ Therefore, the discovery and identification of new and effective drugs that promote synaptic growth are of great clinical importance. As shown in Figure 3b, ISL@PE promoted synaptic growth of NSCs in a manner dependent on time. The percentage of synaptic growth cells demonstrated a significant increase in the ISL@PE, PE, ME and free ISL groups compared to that in control batch (*p* < 0.01) at 72 h. The promotion of synaptic growth of ISL@PE was better than PE and ISL (*p* < 0.01), while no significant promotion could be found in ME group.

### 3.6 Immunomodulatory effects of ISL@PE on microglial cells

**FIGURE 4:**
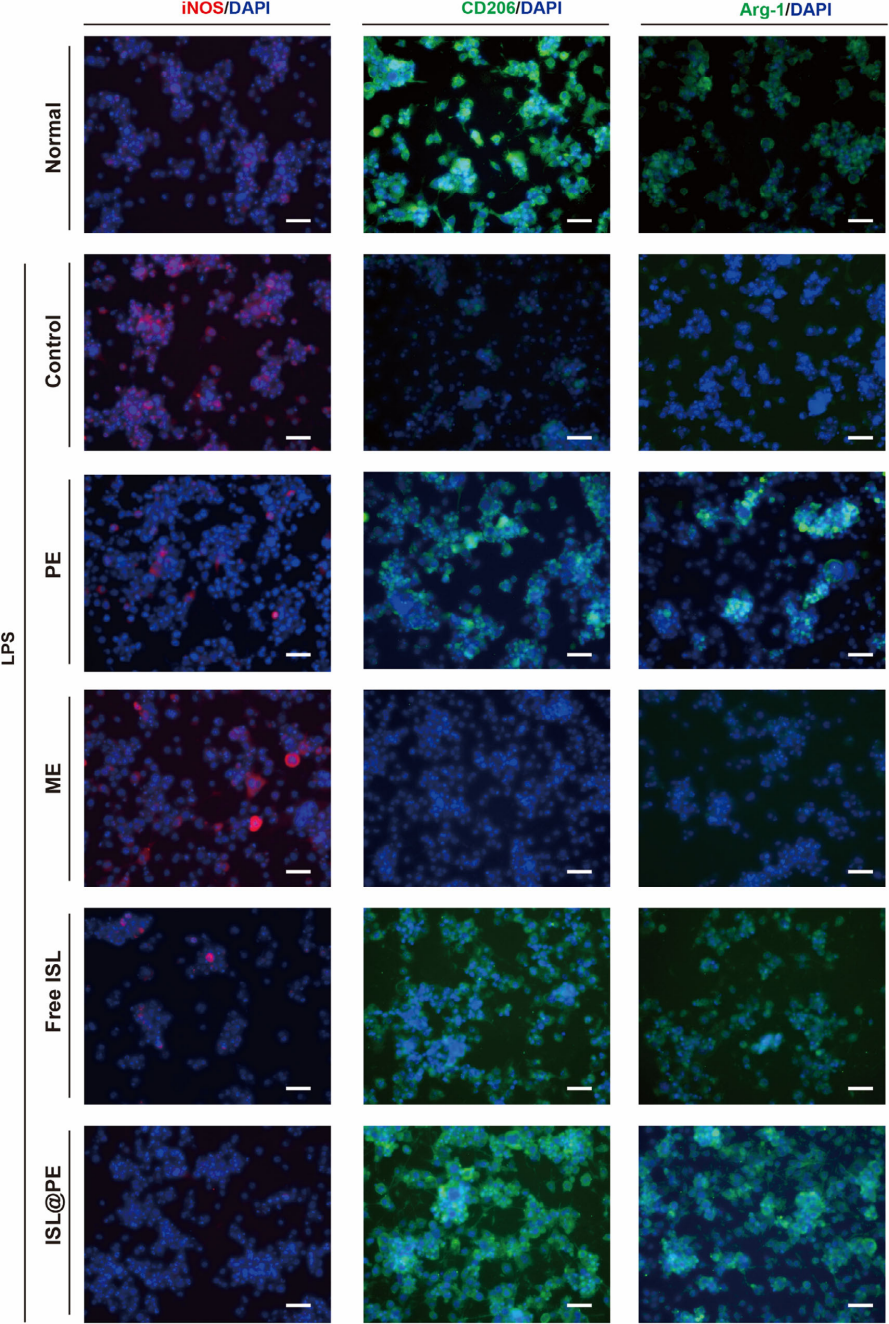
Effect of different groups on polarization of lipopolysaccharide (LPS)‐treated microglial N9 cells. Scale bar equals to 50 μm. Inducible nitric oxide synthase (iNOS) production is an important effect of antimicrobial M1 macrophage activity, whereas anti‐inflammatory (M2) macrophages high expressed arginase‐1 (Arg‐1) and CD206. The LPS‐induced group termed normal group did not express CD206 and Arg‐1, suggesting that the N9 cells could hardly transition from M1 to M2 without intervention. After PE, free ISL, and ISL@PE treatment, N9 cells expressed low levels of the M2 proteins CD206 and Arg‐1, indicating that free ISL and ISL@PE had a beneficial effect on the transition of N9 cells from M1 to M2. No significant changes could be found in ME group.

ISL is a flavonoid derived from licorice, a traditional Chinese medicinal herb, which has neuroprotective effects in damaged PC12 cells.^53^ ISL can inhibit the expression of pro‐inflammatory factors such as COX‐2, iNOS, TNF‐α, and IL‐6, and modulate the polarization of microglia from M1 to M2 phenotype.^54^ ISL is insoluble in water, which limits its solubility and pharmacological activity.^55^ By encapsulating ISL in PEs, we aimed to increase its solubility and enhance its anti‐inflammatory and neuronal differentiation properties. As shown in Figure 4, expression of iNOS increased substantially in LPS‐induced N9 cells. Furthermore, iNOS expression decreased after treatment in the PE, ME, free ISL, and ISL@PE groups. However, the PE and free ISL groups could only inhibit the expression of iNOS to a certain extent. Therefore, the N9 cells still released a considerable amount of pro‐inflammatory factors in these groups. It was observed that iNOS expression in ISL@PE batch was almost completely inhibited. Thus, ISL@PE displayed the strongest inhibitory effect on inflammation relative to all the treatments.

Changes in the N9 cell phenotype were determined with immunofluorescence experiments (Figure 4). Expression of iNOS is used to characterize pro‐inflammatory (M1) macrophages. Production of nitric oxide (NO) is a vital activity of antimicrobial M1 macrophage, whilst NO is not produced by anti‐inflammatory (M2) macrophages. Instead, these immune cells express high Arg‐1 and CD206 proteins levels.^56^ The LPS‐induced group did not express CD206 and Arg‐1, thus suggesting that the N9 cells could hardly transition from M1 to M2 without intervention. After free ISL treatment, N9 cells expressed low levels of the M2 proteins CD206 and Arg‐1, which suggest that free ISL has a beneficial effect on the transition of N9 cells from M1 to M2. The positive expression of CD206 and Arg‐1 in the ISL@PE group indicates that ISL@PE promoted transition from M1‐ to M2‐type in response to LPS stimulation in N9 cells.

**FIGURE 5:**
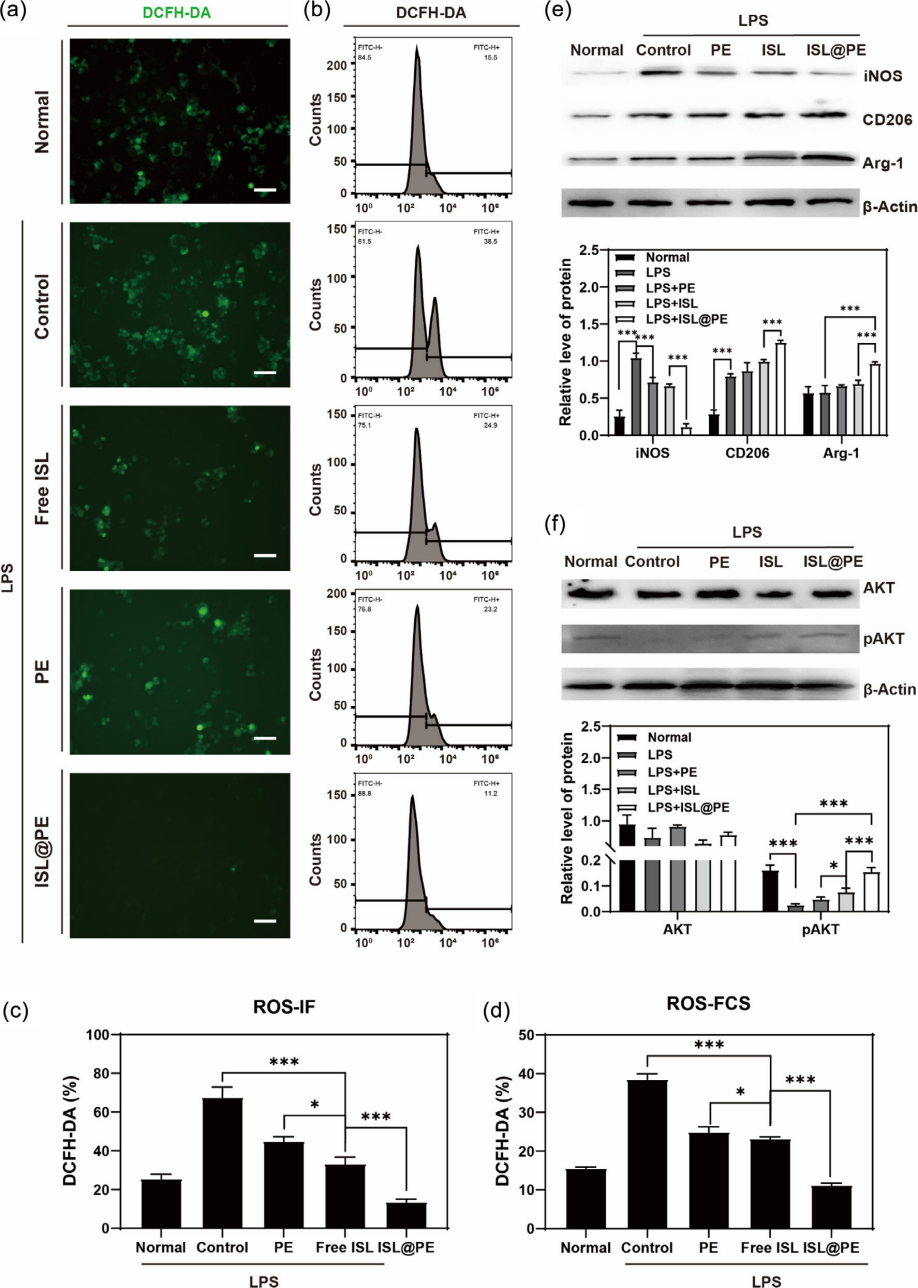
Effect of isoliquiritigenin (ISL)‐loaded plant‐derived exosomes (ISL@PE) on immune reaction in lipopolysaccharide (LPS)‐treated microglial N9 cells. (a) Immunofluorescent staining of oxidative stress in N9 cells. (b) Flow cytometry detection of oxidative stress in N9 cells. (c, d) The statistical results of ROS‐immunofluorescent staining (ROS‐IF) and ROS‐flow cytometry detection (ROS‐FCS). (e) Western blot of iNOS, CD206, and Arg‐1 expression in N9 cells on Day 3 of different treatment groups. (f) Western blot of AKT and pAKT expression in N9 cells on Day 3 of different treatment groups. Scale bars are equal to 50 μm, ***p < 0.01.

As depicted in Figure 5a,b, the production of ROS in the LPS‐induced groups raised substantially (*p* < 0.01) comparable to normal group. Within the LPS‐induced groups, the production of ROS in the free ISL group reduced significantly (*p* < 0.01) compared to PE and control batches. Furthermore, compared to free ISL group, ROS level in the ISL@PE batch decreased substantially (*p* < 0.01) (Figure 5c,d). Inhibition of ROS production in ISL@PE group indicates that ISL@PE inhibited inflammation in vitro. Hence, targeting the M1 to M2 conversion of microglia through inhibition of their mediated neuroinflammatory response is an effective protocol for protecting neurons.

Through results of western blotting (Figure 5e), we observed that iNOS expression in the LPS‐induced groups increased substantially compared to normal group (*p* < 0.01). The expression of iNOS in PE, ISL, and ISL@PE groups decreased significantly (*p* < 0.01) comparable to control LPS‐induced batch, amid the ISL@PE group showing the lowest level of iNOS protein expression. In contrast, M2 cell markers (CD206 and Arg‐1) expression in LPS‐induced group decreased markedly compared to normal group (*p* < 0.01). In comparison with control LPS‐induced batch, CD206 and Arg‐1 expressions in ISL@PE group increased remarkably (*p* < 0.01), thus indicating that ISL@PE increased CD206 and Arg‐1 proteins expressions when induced by LPS.

Scientists have observed the involvement of AKT in inflammation, wherein it is a key protein in PI3K downstream signaling pathway.^57^ The LPS stimulate human intrinsic immune cells.^58^ Additionally, IL‐6, IL‐12, and TNF‐α (pro‐inflammatory factors) expression increases whilst that of IL‐10 (anti‐inflammatory factor) decreases after the application of PI3K or AKT inhibitors.^59^ As shown in Figure 5f, AKT expression was not substantially different within groups. Nonetheless, expression of pAKT/AKT in LPS‐induced groups reduced remarkably compared to control group (*p* < 0.01). In comparison with LPS‐induced control group, the expression of pAKT in N9 cells of PE, ISL, and ISL@PE groups was significantly higher (*p* < 0.01), with the highest pAKT expression found in ISL@PE batch. This indicated that both PE and ISL increased expression of pAKT protein in LPS‐induced N9 cells, while ISL@PE showed best combined effect. Thus, we can preliminarily conclude that PE and ISL can convert pro‐inflammatory N9 cells into anti‐inflammatory cells. Formulation ISL@PE showed the strongest suppression of inflammation by superimposing the effects.

### 3.7 SCI repair by 3D‐printed hydrogel scaffold containing ISL@PE

**FIGURE 6:**
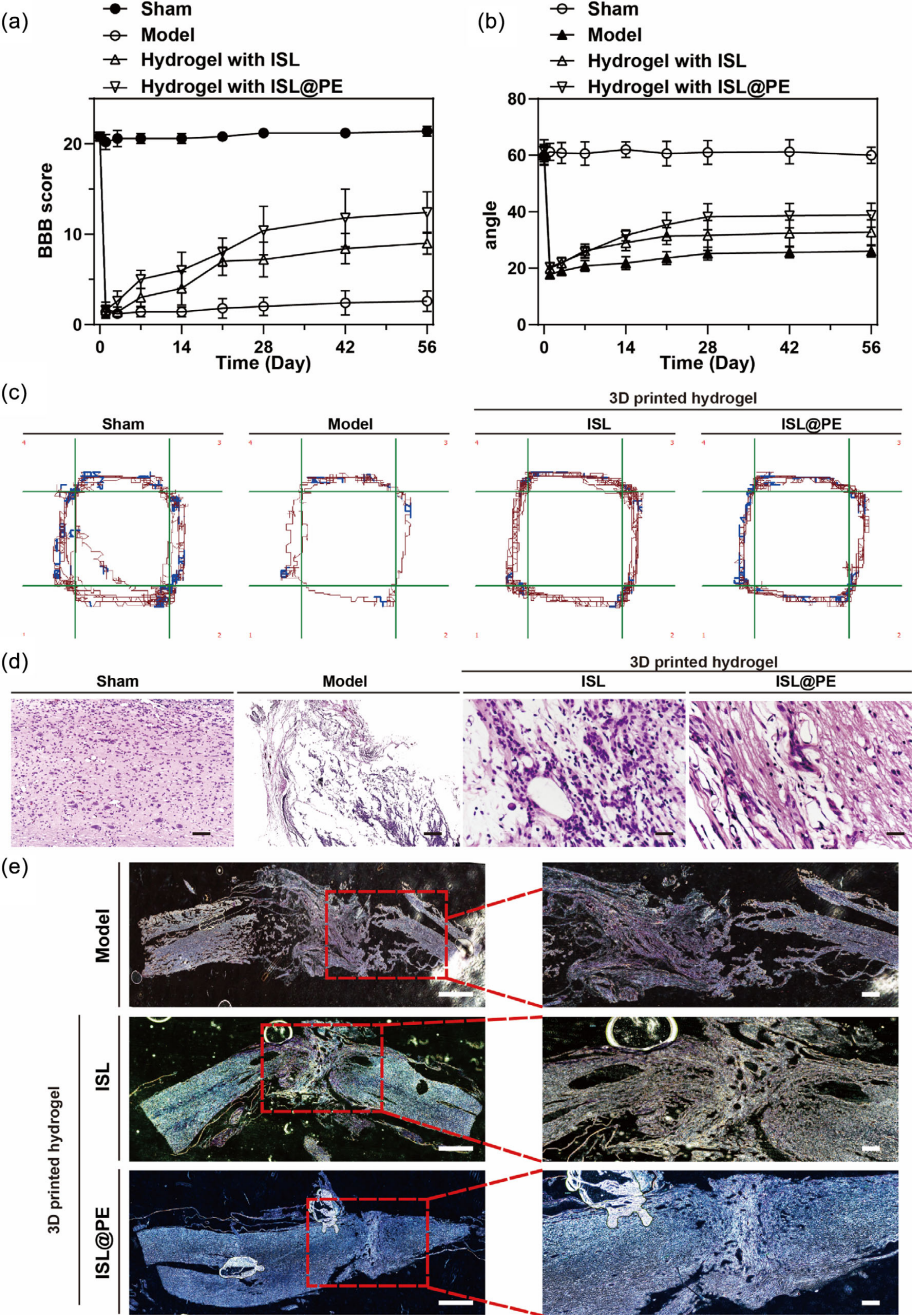
The positive effect of 3D‐printed hydrogel with isoliquiritigenin (ISL)‐loaded plant‐derived exosomes (ISL@PE) in treating spinal cord injury. (a) Basso–Beattle–Bresnahan (BBB). (b) Inclined plane test. (c) Open field test. (d) Nissl staining. (e) HE staining. Scale bar equals to 50 μm.

We established a rat SCI model via amputation of spinal cord and implanted a 3D printed hydrogel in injured segment of spinal cord. Behavioral changes in rats after SCI repair were observed using BBB scores, inclined plate tests, and open field tests. Comparison of multiple tests proved that the behavioral evaluations were consistent.^60^ We observed higher BBB and open‐field scores in ISL@PE compared to SCI group at 7, 14, 21, 28, 42, and 56 days (Figure 6a). During 2 weeks of feeding, the hindlimbs of the three injured groups could move slightly, while the BBB scores were similar among the three groups, albeit insignificantly different. After 4 weeks of feeding, the SCI control (model) group showed poor recovery of motor function but its BBB score remained at a low level with a final value of 2.5 ± 0.5. After 4 weeks of feeding, the BBB score of the implanted ISL scaffold group showed an increase until the 8th week with a final score of 8.0 ± 0.82. The motor function of these rats improved slightly. However, they could not support their weight while standing, amid their movements being uncoordinated. Finally, the BBB score of the implanted ISL@PE stent group increased substantially compared to the model batch, but the rats could support their weight while standing and walking.

Through an inclined plane experiment (Figure 6b), we demonstrated that the critical angle of rats in the normal group was approximately 60°, while that of the rats in model group decreased sharply at 1 week, amid a gradual recovery overtime, which was approximately 30° by the 8th week. In contrast, the critical angle of both hydrogel groups was improved comparable to that of model batch, with the ISL@PE group showing a significant difference.

An open‐field experiment was conducted to examine rats' recovery after SCI by detecting their movements in a natural state.^61^ The results of 8‐week open field experiments in different groups showed that the total distance moved in the 3D printing batch was greater compared to SCI model group (*p* < 0.01). Likewise, the total distance of rats moved in an open field in the ISL@PE group after surgery was more complex comparable to the ISL batch (Figure 6c), which suggests that the ISL@PE group showed the best repair effect.

Furthermore, HE and Nissl staining were used to examine the spinal tissues histologically. Eight weeks after surgery, the damaged spinal cord tissues of each group were observed microscopically using HE staining. The results showed that the spinal nerves in 3D‐printed hydrogel groups regrew well, whereas those in the model group were damaged, thus showing evidence of edema, sparse cells, and increased intercellular gaps (Figure 6d). In the ISL group, cell growth was tighter than in model group, while vacuoles' number was reduced. The spinal nerves regrew well in the ISL@PE batch, while the gaps between the tissues were narrowed, coupled with reduced vacuoles and disappearance of tissue edema.

**FIGURE 7:**
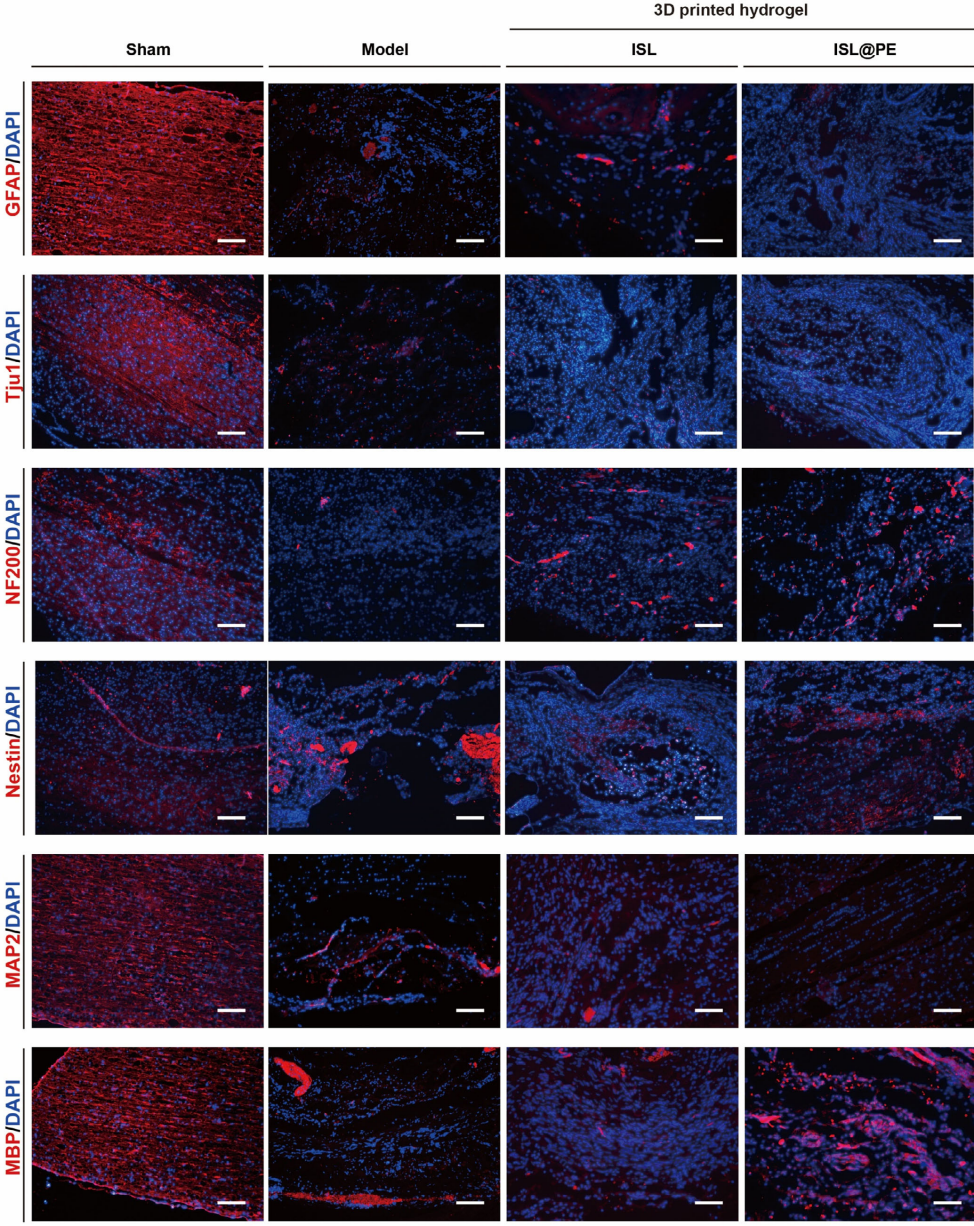
Fluorescent staining of GFAP, Tuj1, NF200, Nestin, MAP2, and MBP proteins in the injured spinal cord of different treatment groups. Scale bars are equal to 50 μm. A positive expression of Tuj1, NF200, Nestin, MAP2, and MBP could be found in 3D‐printed hydrogel group with isoliquiritigenin (ISL)‐loaded plant‐derived exosomes (ISL@PE) compared to the model group.

Nissl staining showed that the number of motor neurons in the caudal ventral horn of spinal cord was decreased to varying degrees in other groups comparable to sham‐operated batch. At 8 weeks post‐operation, we observed more motor neurons in ventral horn of spinal cord in hydrogel groups compared to SCI model batch (*p* < 0.05). Furthermore, those neurons in the ISL@PE group increased substantially comparable to SCI model group (*p* > 0.05). The number of motor neurons in the caudal anterior horn of spinal cord in SCI area increased in the ISL@PE batch compared to SCI model group (*p* < 0.05; Figure 6e). Immunofluorescent staining demonstrated reduced expression of neural markers related to SCI area to distinct degrees in the 3D‐printed hydrogel groups at 8 weeks after surgery (Figure 7) compared to the sham‐operated group. Notably, the expression of neural markers increased in the ISL@PE batch compared to ISL group.

**FIGURE 8:**
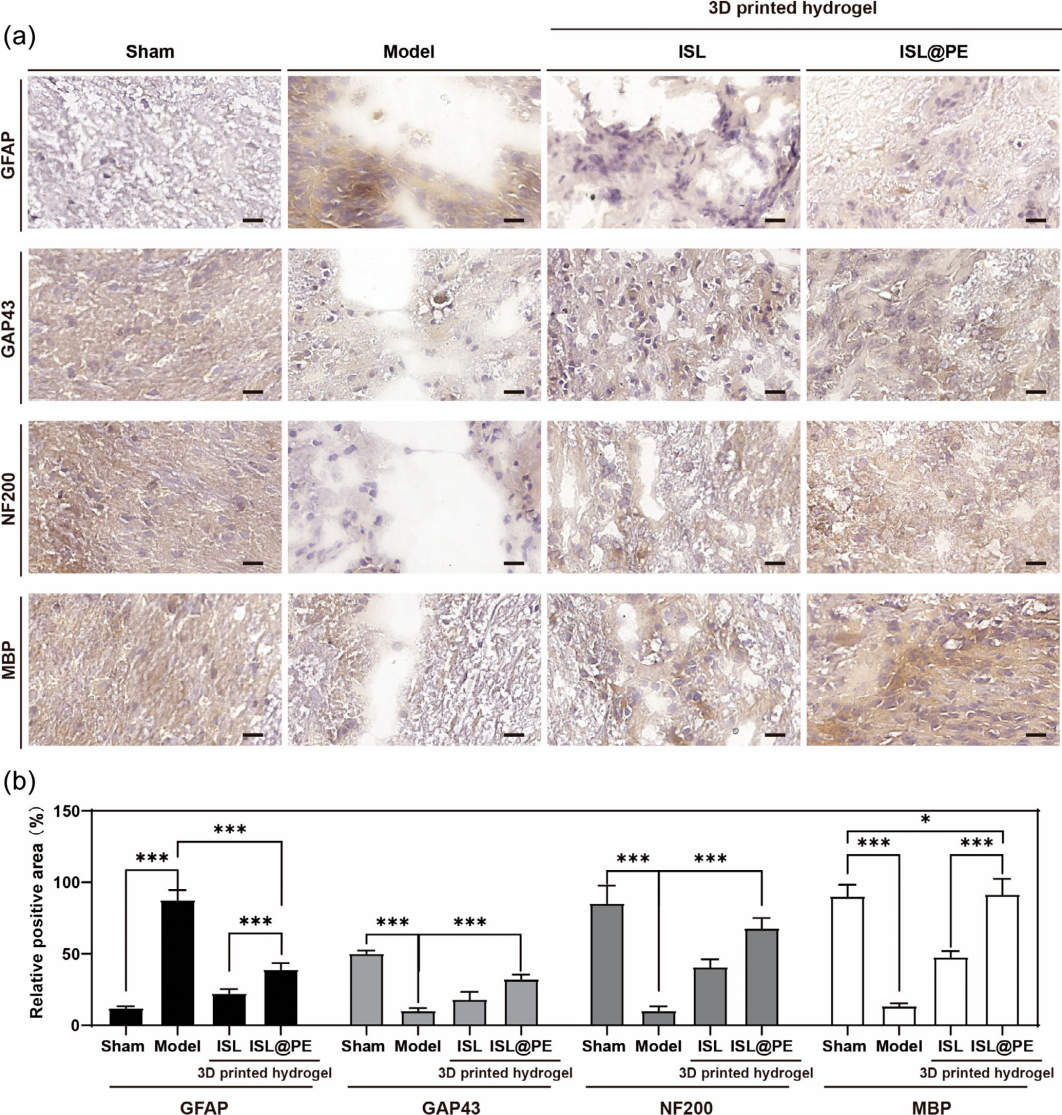
Immune staining of GFAP, GAP43, NF200, and MBP proteins of the injured spinal cord in different treatment groups. (a) Immunohistochemical staining of relative protein expressed in different group. (b) Quantitative analysis of immunohistochemical staining. There is a higher expression of GAP43, NF200, and MBP in the spinal cord 3D printed hydrogel containing isoliquiritigenin (ISL)‐loaded plant‐derived exosomes (ISL@PE) compared to that containing ISL. Scale bar equals to 50 μm. ***p < 0.01.

To further quantify positive effect of the 3D‐printed hydrogel containing ISL@PE, we detected the related neural markers by IHC staining before their quantification with Fiji software. As shown in Figure 8a, by comparing the corresponding parts of spinal cord after 8 weeks in each group, it was found that the staining of GAP43, NF200, and MBP proteins increased significantly in the 3D‐printed hydrogel groups. The percentage of positive particles counted under high magnification was markedly higher compared to other groups (*p* < 0.01; Figure 8b), thus indicating significant increased expression of the three upregulated proteins. The trends of the three upregulated proteins in the 3D‐printed hydrogel groups were consistent with the differences obtained from previous immunofluorescence studies. Meanwhile, the results showed that identification of the three upregulated proteins and changes in their expression were accurate. IHC analysis revealed the most significant changes in expression in the ISL@PE group compared to other groups. An opposite trend was observed for GFAP expression.

GAP43, a neuron‐specific presynaptic membrane protein^62^ enables neurons to send out new terminals, even in the absence of other trophic factors, and regulates synaptic extension and plasticity. Scientists have established the significant role of myelin basic protein (MBP) in myelin formation in nerve cells.^63^ As a main constituent of myelin sheath that surrounds axons, MBP contributes to cytoplasmic membrane that adheres to tightly packed myelin. This essentially facilitates the conduction of neuronal impulses. Another study on protein changes in SCI tissues found that GFAP protein expression increased significantly post injury, thus demonstrating that it is a marker of glial scar tissue.^64^ Therefore, combining the results of the experimental and IHC studies, we believe that the implantation of a 3D‐printed scaffold containing ISL@PE in the acutely injured segmental spinal cord of rats may serve to promote repair and regeneration of damaged neuronal cells, thus achieving repair of the spinal cord.

### 3.8 Mechanism of SCI repair in vivo

**FIGURE 9:**
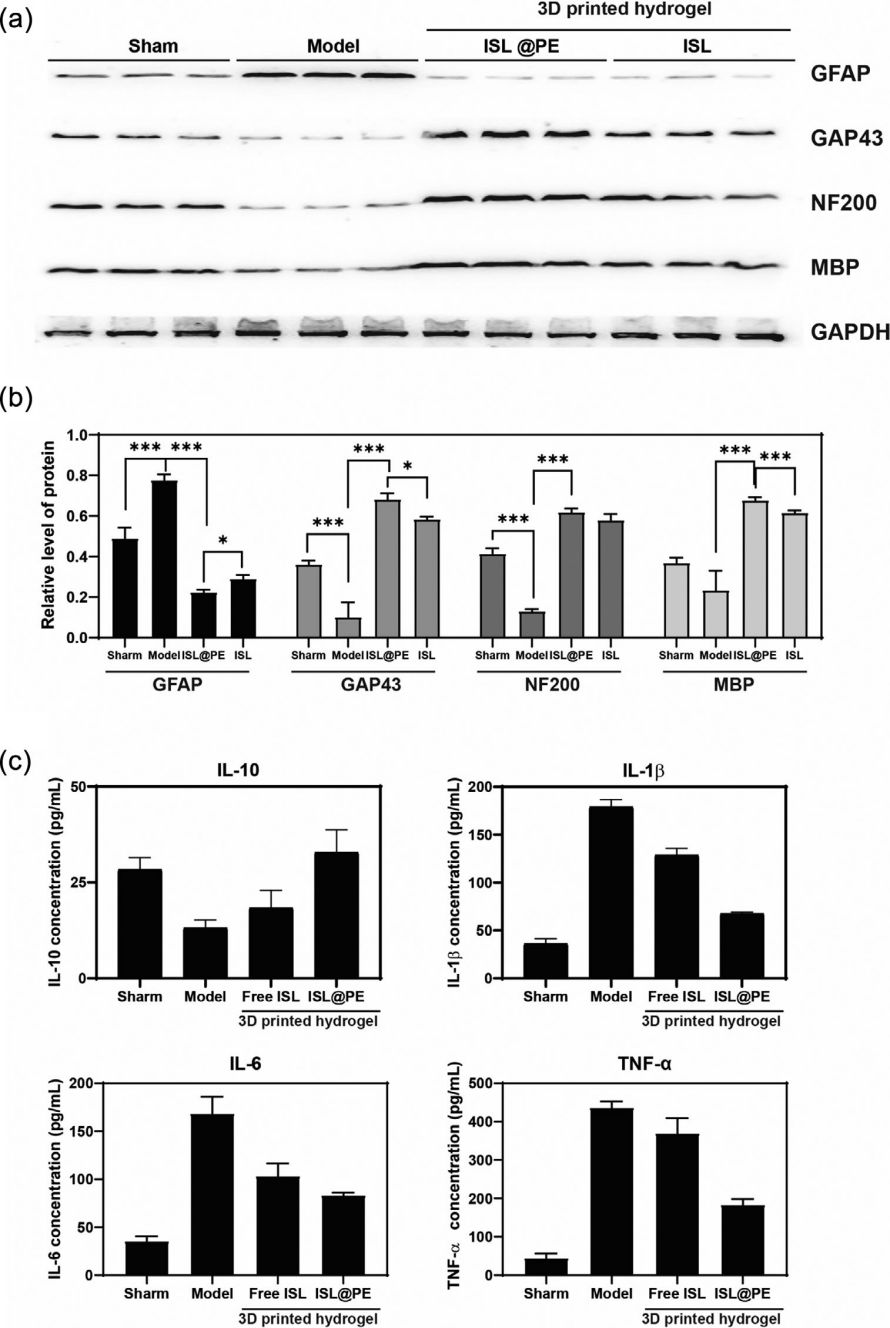
Relative expression of GFAP, GAP43, NF200, MBP, and GAPDH proteins in the injured spinal cord in different treatment groups. Western Blot protein expressions (a) and quantification data (b) of GFAP, Tuj1, NF200, Nestin, MAP2, and MBP in each group. (c) Quantification data of IL‐10, IL‐1β, IL‐6, and TNF‐α of injured spinal cord in different treatment groups. ***p < 0.01.

To further examine the effect of 3D‐printed hydrogel scaffold, we collected the injured segment of spinal cord in different groups. Western blotting results (Figure 9a,b) demonstrated increased levels of GAP43, NF200, and MBP expression in the 3D‐printed hydrogel groups comparable to those in model batch (*p* < 0.05), while GAP43 and MBP levels in ISL@PE group up‐regulated compared to ISL batch (*p* < 0.05). Also, we observed a substantial lowered expression of GFAP in 3D‐printed hydrogel group compared to model group, thus indicating that the 3D‐printed scaffold containing ISL@PE promoted nerve regeneration and inhibited scar tissue formation.

ELISA results (Figure 9c) showed that the expression of pro‐inflammatory cytokines (IL‐1β, IL‐6, and TNF‐α) at protein levels in injured spinal cord segment of the 3D‐printed hydrogel groups decreased substantially compared to model group (*p* < 0.01), but an increased IL‐10 expression in 3D‐printed hydrogel groups was observed comparable to model batch. The expression of above‐mentioned pro‐inflammatory cytokines at protein levels in ISL@PE group reduced obviously compared to ISL group (*p* < 0.01). This result suggests that ISL@PE could attenuate inflammatory response in vivo.

## 4 CONCLUSIONS

To address the processing demand for therapeutic strategies that enhance SCI repair, we devised composite hydrogel scaffolds employing 3D printing with biomimetic architectures, incorporating PEs from *L. barbarum* L. We successfully encapsulated isoliquiritigenin (ISL) in PE (ISL@PE) and ascertained that both PE and ISL exhibited anti‐inflammatory and neurogenic differentiation properties in vitro, with ISL@PE manifesting a synergistic effect. Our examination of pAKT/AKT pathway revealed that the inflammation activation mechanism might be attained by increased pAKT expression. Furthermore, our assessment of the effect of 3D‐printed complex on SCI repair and its mechanism through in vivo experimentations indicated that the scaffold could effectively promote recovery of motor function, reduce glial scarring, and inhibit inflammatory responses in rats. These findings substantiated the positive impact of the 3D‐printed scaffold combined with ISL and PE on inflammation abatement and nerve regeneration in SCI. To summarize, our investigation highlights the potential of composite hydrogel scaffolds with bionic structures containing ISL@PE, as a promising approach for SCI treatment. Additional research is needed to thoroughly elucidate the underlying mechanisms and optimize the scaffold's design and composition.

## AUTHOR CONTRIBUTIONS

**Qilong Wang:** Conceptualization (equal); methodology (equal); resources (lead); supervision (equal). **Kai Liu:** Data curation (equal); formal analysis (equal); writing – original draft (equal). **Xia Cao:** Conceptualization (equal); funding acquisition (equal); project administration (equal); supervision (equal). **Wanjin Rong:** Formal analysis (equal); investigation (equal); methodology (equal); software (equal); validation (equal). **Wenwan Shi:** Data curation (equal); formal analysis (equal); investigation (equal); methodology (equal); resources (equal). **Qintong Yu:** Data curation (equal); formal analysis (equal); validation (equal). **Wenwen Deng:** Formal analysis (equal); investigation (equal). **Jiangnan Yu:** Funding acquisition (equal); supervision (equal). **Ximing Xu:** Funding acquisition (equal); project administration (equal); supervision (equal).

## FUNDING INFORMATION

This work was funded by the National Key R&D Program of China (2018YFE0208600), National Natural Science Foundation of China (81720108030 and 82173785), Key Planning Social Development Projects of Zhenjiang in Jiangsu Province (SH2021024), Natural Science Foundation of Jiangsu Province (BK20220529), Postdoctoral Research Fund of Jiangsu Province in 2021 category A (2021K010A), and the financial support by Key Lab for Drug Delivery & Tissue Regeneration (SS2018004).

## CONFLICT OF INTEREST STATEMENT

The authors declare no conflicts of interest.

### PEER REVIEW

The peer review history for this article is available at https://www.webofscience.com/api/gateway/wos/peer-review/10.1002/btm2.10646.

## Data Availability

The authors confirm that the data supporting the findings of this study are available within the article.
